# All-inside versus inside-out suture techniques in arthroscopic meniscus repair: A prospective randomized study protocol

**DOI:** 10.1097/MD.0000000000020688

**Published:** 2020-07-02

**Authors:** Yanming Lin, Jiasong Zhao, Heng Qiu, Yong Huang

**Affiliations:** Department of Orthopaedics, Hospital of Chengdu University of Traditional Chinese Medicine, Sichuan 610072, China.

**Keywords:** all-inside, arthroscopic meniscus repair, inside-out, randomized controlled trial, study protocol

## Abstract

**Background:**

With advancements in our understanding of meniscal function, treatment options for meniscal injuries have evolved considerably over the past few decades. The aim of the current study was to compare the all-inside and inside-out techniques with regard to retear rate, functional outcomes, and perioperative complications in patients who had undergone arthroscopic meniscus repair. We hypothesized that there was no significant difference between the 2 groups in terms of postoperative outcomes after arthroscopic meniscus repair.

**Methods:**

This study was a prospective randomized blinded study, with a parallel design and an allocation ratio of 1:1 for the treatment groups. This study was approved by the Institutional Review Board in our hospital and written informed consent was obtained from all subjects participating in the trial. It was carried out in accordance with the principles of the Helsinki Declaration. A total of 70 patients who meet inclusion criteria are randomized to either all-inside or inside-out group. The primary outcome measure was retear rate. Retear was determined by repeat arthroscopic evaluation of patients with follow-up for symptoms of persistent or new pain, catching, or locking that was possibly related to the meniscal repair. Secondary outcomes included disease-specific quality of life measurement with the Western Ontario Meniscal Evaluation Tool, range of motion, operative time, and adverse events at surgery or throughout the follow-up period.

**Results:**

This study has limited inclusion and exclusion criteria and a well-controlled intervention.

**Trial registration:**

This study protocol was registered in Research Registry (researchregistry5589).

## 1 Introduction

With advancements in our understanding of meniscal function, treatment options for meniscal injuries have evolved considerably over the past few decades. The importance of the menisci in shock absorption, stability, and load transmission has pushed orthopaedists to transition from meniscal removal to preservation procedures, particularly in instances where repair may be successful.^[1–3]^ More recently, the use of arthroscopic techniques has gained favor compared with open meniscal repair. Compared with open meniscus surgery, arthroscopic meniscus surgery has various beneficial effects such as short operation time, early recovery, and minimal trauma. Despite these benefits, arthroscopic meniscectomy can cause disruption of the circumferential fibers, which can ultimately lead to the inability of the remaining meniscus to effectively control hoop stress.^[4–6]^

The inside-out repair technique has been used commonly for posterior horn meniscal tears. However, this technique requires an additional skin incision and has potential for neurovascular complications and postoperative stiffness.^[7]^ At present, arthroscopic meniscal repair has evolved from inside-out to all-inside repair technique. The all-inside technique is currently the method of choice for many surgeons and was developed to reduce surgical time, allow access to the posterior horns of the menisci, and prevent complications that occur with the external approach.^[8]^ The techniques used for this method have evolved; earlier generation repairs involved the use of curved suture hooks and self-adjusting suture devices. More recent generations are aimed at reducing the need for additional incisions and include the use of arrows, screws, and anchors, intended to improve compression and tensioning.^[9]^

However, very few studies comparing the inside-out technique with the all-inside meniscal repair systems are available in the literature in order to draw useful conclusions. The aim of the current study was to compare the all-inside and inside-out techniques with regard to retear rate, functional outcomes, and perioperative complications in patients who had undergone arthroscopic meniscus repair. We hypothesized that there was no significant difference between the 2 groups in terms of postoperative outcomes after arthroscopic meniscus repair.

## 2 Materials and methods

This study was a prospective randomized blinded study, with a parallel design and an allocation ratio of 1:1 for the treatment groups. This study was approved by the Institutional Review Board in our hospital (CDH2020347) and written informed consent was obtained from all subjects participating in the trial. The trial was also registered at the Research Registry (researchregistry5589). It was carried out in accordance with the principles of the Helsinki Declaration. Data are presented according to the CONSORT statement.

### 2.1 Patients

The inclusion criterion were set as follows:

(1) Inclusion criteria were patients with longitudinal meniscal tears in the red-red (<3 mm from the synovial meniscal junction) or red-white (3–5 mm from the synovial meniscal junction) zones of the meniscus;
(2) patients that were over 18 years old and could cooperate with us for treatment and postoperative observation;
(3) American Society of Anesthesiologists status of I to III.

Exclusion criteria included: patients had undergone a previous meniscal repair in the operative knee, had an unstable knee joint and were not undergoing concomitant ligament reconstruction, or had active joint or systemic infection, or had a major medical illness that would preclude surgery. Other exclusions included those patients who were unwilling or unable to be followed according to study protocol for 2 years, including patients who had plans to move outside of the vicinity of the participating center; those who had a major psychiatric illness; those who were intellectually challenged; and those unable to speak or understand the Chinese language.

### 2.2 Randomization

**Figure 1:**
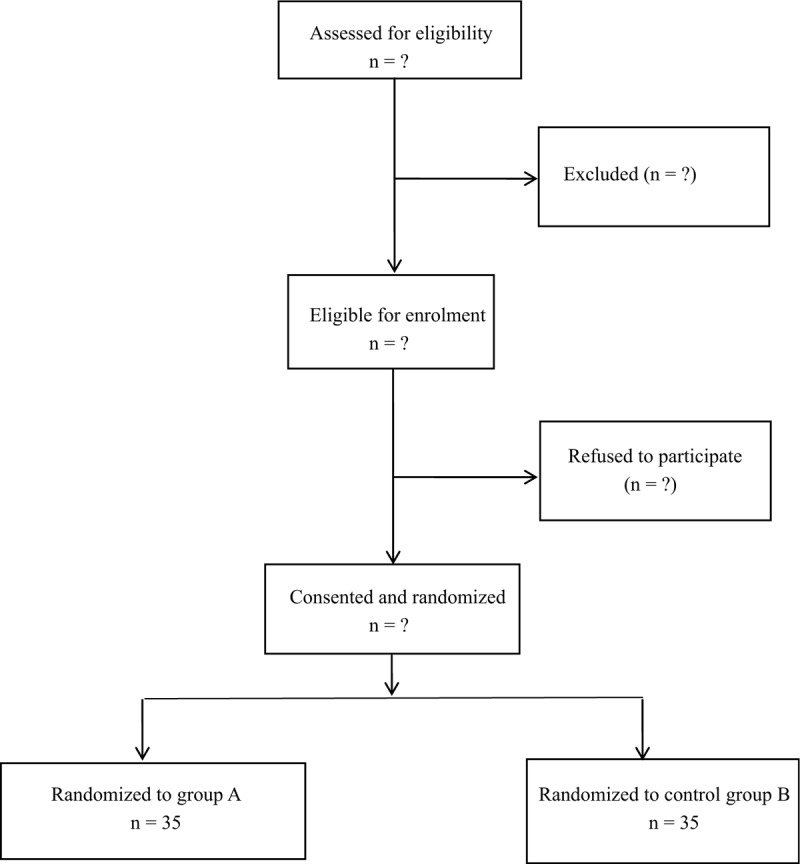
Flow of patients through the trial.

An equal number of envelopes for each treatment group were prepared using a computerized random number generator by a study assistant who did not take part in any subsequent part of the study, and was not in contact with the rest of the study team throughout the entire study duration. He prepared 70 identical sequentially numbered, opaque, sealed, and stapled envelopes; 35 envelopes contained instructions for mixing solutions for Group A (all-inside), and the other 35 for Group B (inside-out). The envelopes were kept in a file with the principal investigator (Fig. 1).

### 2.3 Surgical techniques

#### 2.3.1 All-insight group

In patients who underwent all-inside meniscal repair, the 70° arthroscope was placed from the anterolateral portal into the posteromedial compartment through the intercondylar notch. An 18-gauge spinal needle punctured the posteromedial capsule, and an 8-mm skin incision was made; an 8.5-mm disposable cannula was placed. A motorized shaver was inserted through the cannula to abrade the meniscal tear site to enhance healing. A curved suture hook was inserted through the cannula and penetrated the inner rim of the meniscus from superior to inferior and then penetrated the outer rim from inferior to superior. The leading limb of a No. 1 polydioxanone suture was advanced sufficiently into the joint, and the suture hook was retrieved. The leading limb was retrieved and the sutures tied through the cannula. Sutures were placed every 5 mm along the length of the tear.

#### 2.3.2 Inside-out group

In patients who underwent inside-out meniscal repair, a 3-cm skin incision was made at the postero-medial border of the proximal tibia just below the joint line. The sartorius muscle fascia was opened, and the gracilis and semitendinosus tendons were retracted posteriorly. The medial head of the gastrocnemius muscle was retracted posteriorly, and the posterior capsule of the knee was palpated. A right-angled meniscal rasp was inserted through the anteromedial portal and used to abrade the meniscal tear site to enhance healing. A zone-specific meniscal repair cannula was placed on the upper surface of the meniscus, and a Nitinol needle was used to pass polydioxanone sutures, placed in vertical mattress fashion. Sutures were placed every 5 mm along the length of the tear. Sutures were tied securely over the posteromedial capsule of the knee.

### 2.4 Postoperative care

Patients who underwent an isolated meniscal repair had the knee locked in extension using a splint for 3 weeks after surgery and were allowed protected weight-bearing (full weightbearing with the protection of crutches). After 3 weeks, weightbearing was as tolerated, and range of motion was unlimited. Patients were instructed to avoid squatting, pivoting, and twisting for a minimum of 6 months.

### 2.5 Outcome measures

The primary outcome measure was retear rate. Retear was determined by repeat arthroscopic evaluation of patients with follow-up for symptoms of persistent or new pain, catching, or locking that was possibly related to the meniscal repair. Secondary outcomes included disease-specific quality of life measurement with the Western Ontario Meniscal Evaluation Tool (WOMET), range of motion (ROM), operative time, and adverse events at surgery or throughout the follow-up period.

The WOMET is a validated, reliable, and responsive patient-based 16-item questionnaire (100-mm visual analog scale response format), which inquires into the domains of physical symptoms, sports/recreation/work/lifestyle, and emotional well-being. This questionnaire provides a subjective measure of quality of life for patients with meniscal injury. A patient's score is determined by calculating the sum of each domain to attain a total score out of 1600, which is then converted to a mean score of 100, with 100% being the best possible score. The ROM were measured by the research assistant for both knees with a standard universal goniometer, which has been shown to have good intraobserver and interobserver reliability in the knee joint.

### 2.6 Statistical analysis

Statistical analyses were conducted using SPSS v22.0 software (IBM, Chicago, IL). Conformity of the data to normal distribution was tested with the Kolmogorove–Smirnov test. Independent 2 samples *t*-test was used for comparison of continuous variables and Pearson Chi-square test was used for comparison of categorical variables. Results were evaluated in a confidence interval of 95% and at a significance level of *P* < .05.

## 3 Discussion

Meniscal repair is preferable to partial meniscectomy based on documented healing of tears by second-look arthroscopy and prevention of radiographic changes of early osteoarthritis in long-term studies. There are 2 arthroscopic techniques for meniscal repair: the inside-out and the all-inside technique. The use of all-inside meniscal repair systems has been increasing dramatically in the last years mainly because it is technically less demanding and easier for the surgeon in comparison with suturing methods. Furthermore, few studies in literature has studied the differences in efficacy and safety of the all-inside and inside-out techniques after arthroscopic meniscal repair.^[10–12]^ Therefore, the purpose of this study was to compare the effectiveness of all-inside and inside-out techniques for meniscal repair.

Three potential limitations to this study were identified. First, the subjects may be exclusively Chinese. Therefore, the data from this clinical trial cannot be applied to other ethnic groups. The second limitation was the small sample size used in this study. Replication of this study on a multi-institutional level would provide less variation between experimental groups and simultaneously increase the reliability and generalizability of the results. Finally, having the same surgeon for all procedures in this study was both an advantage and disadvantage. It is advantageous for consistency and internal validity; however, it may limit the reproducibility of this study.

## Author contributions

**Conceptualization:** Yanming Lin.

**Data curation:** Jiasong Zhao.

**Formal analysis:** Yanming Lin, Jiasong Zhao.

**Funding acquisition:** Yong Huang.

**Investigation:** Yanming Lin, Jiasong Zhao.

**Methodology:** Yanming Lin.

**Resources:** Yong Huang.

**Software:** Heng Qiu.

**Supervision:** Yong Huang.

**Validation:** Heng Qiu.

**Visualization:** Heng Qiu.

**Writing – original draft:** Yanming Lin, Jiasong Zhao

**Writing – review & editing:** Yong Huang.
